# Photo-Induced Black Phase Stabilization of CsPbI_3_ QDs Films

**DOI:** 10.3390/nano10081586

**Published:** 2020-08-12

**Authors:** Eider A. Erazo, H.E. Sánchez-Godoy, Andrés F. Gualdrón-Reyes, Sofia Masi, Iván Mora-Seró

**Affiliations:** 1 Institute of Advanced Materials (INAM), Universitat Jaume I (UJI), Avenida de Vicent Sos Baynat, s/n, 12071 Castellón de la Plana, Spain; ea.erazo@uniandes.edu.co (E.A.E.); Humberto.Sanchez@academico.udg.mx (H.E.S.-G.); gualdron@uji.es (A.F.G.-R.); 2 Departamento de Química, Universidad de los Andes, Bogotá D.C. 111711, Colombia; 3 Centro Universitario de los Lagos, Universidad de Guadalajara, Lagos de Moreno, Jalisco C.P. 47460, Mexico

**Keywords:** CsPbI_3_ QDs, stability, solar cells, UV photo-induced stabilization

## Abstract

α-CsPbI_3_ quantum dots (QDs) show outstanding photoelectrical properties that had been harnessed in the fabrication of perovskite QDs solar cells. Nevertheless, the stabilization of the CsPbI_3_ perovskite cubic phase remains a challenge due to its own thermodynamic and the presence of surface defects. Herein, we report the optimization of the CsPbI_3_ QDs solar cells, by monitoring the structure, the morphology and the optoelectronic properties after a precise treatment, consisting of the conventional solvent washing with a time limited ultraviolet (UV) exposure combination, during the layer-by-layer deposition. The UV treatment compensates the defects coming from the essential but deleterious washing treatment. The material is stable for 200 h and the PCE improved by the 25% compared with that of the device without UV treatment. The photo-enhanced ion mobility mechanism is discussed as the main process for the CsPbI_3_ QDs and solar cell stability.

## 1. Introduction

Perovskite solar cells are the most promising next generation photovoltaic technologies to produce solar energy with very high efficiency [1]. Currently, the main limitation is the perovskite low stability in ambient conditions [2]. The halide ABX_3_ perovskite materials are based on two cations, A (formamidinium, methylammonium, and Cs), a metallic one B (Pb^2+^, Sn^2+^), and the halide X (I^−^, Br^−^, Cl^−^) [3], where the *a priori* most stable perovskite, with a suitable band gap (1.73 eV) [4] for photovoltaic application, is CsPbI_3_ due to its inorganic nature. The polymorphic behavior of the perovskite causes the thermodynamic instability of the perovskite that is characterized by a black α-phase, with a cubic or tetragonal crystal structure, predominant only at high temperature [2]. Otherwise it converts in a non-perovskite yellow δ-phase, with an orthorhombic crystal structure. An effective approach to increase the α-phase stability is the manipulation of the dimensionality, moving from the 3D (bulk material) to the quantum dots (QDs) [2,5], with higher phase stability than the bulk counterpart, mainly due to the increase of the surface energy. Perovskite QDs used as active materials in a solar cell led to a photoconversion efficiency (PCE) similar to the one obtained with the bulk perovskite, 16.6%; it has been achieved with a mixed perovskite Cs_1−x_FA_x_PbI_3_ QDs [6], with the benefit that the low dimensionality and the combination of the two cations (cesium and formamidinium) for tuning the tolerance factor, definitively improve the stability in air [7]. Perovskite QDs present very high photoluminescence quantum yield (PLQY) indicating a reduced non-radiative recombination, allowing very high open circuit voltage (V_oc_) [8]. The reduced phase segregation in mixed halide perovskite nanoparticles allows for the real tuning of the perovskite bandgap with important implications for the development of tandem devices [9,10,11,12].

To take advantage of these properties for the development of perovskite quantum dots (QDs) solar cells (PQDSCs), a proper QDs coupling is needed to obtain good transport properties in QDs thin films, with a good carrier collection efficiency, essential for a working device [8]. In order to get it, a fraction of organic capping ligand, covering the colloidal QDs surface has to be removed by specific treatments. This strategy allows for closer QDs and improved transport properties of the QDs thin film. Thus, these treatments lead to two important consequences: i) the reduction of the spatial distance between the QDs and in turn the improving of the electronic coupling; ii) after removing the ligand, the QDs are not more soluble in the original solvent (i.e., octane) and another layer can be deposited on the top. It makes make possible the preparation of thicker films, necessary to increase the light absorption. Repeating the deposition-treatment steps, thicker films can be obtained by a layer-by-layer deposition, allowing for thickness control and for preparing mixed material films, stacking different types of perovskite QDs [13].

The solvent choice used to remove the ligand and improve the QD-to-QD coupling is limited to an organic non hydrogenated solvent, such as methylacetate or ethylacetate, due to the chemical compatibility with the oleate ligand and to the requirement of preserving the perovskite material itself. During this step, a ligand exchange mechanism establishes the replacement of the oleate ligand by acetic acid, resulted from the hydrolysis of methyl/ethyl acetate in air, releasing the free oleic acid [12,14]. Moreover, it has been demonstrated that this step has a huge potential, since this strategy mediates the passivation of vacancies [14], and increases the stability of the CsPbI_3_ when the effect of these solvents is aided by additives or additional treatments [11]. For instance, after the addition of an organic molecule such as the phenylethylammonium iodide (PEAI), the additive remains outside of the perovskite structure and the optical properties of the CsPbI_3_ are preserved. The PCE of solar cells by using this method is about 6%, stable for 4 months, demonstrating that the synergistic control of the surface energy and the surface functionalization has an important role in exceeding the stabilization limit [15]. Despite the effectiveness of the method, the presence of organic molecules in the active layer, that are soluble in the common solvent used for the deposition of the hole transporting material (HTM) (i.e., the chlorobenzene) could lead to an infiltration inconvenience. So far, this problem limits the performances due interface losses or more drastically, for the complete bleaching of the affected perovskite. Another approach, avoiding the use of organic additives, is the optimization of the bottom interface. The treatment with inorganic salts of the bottom layer (i.e., the mesoporous TiO_2_) leads to promising results, as the PCE increased from 5.58% to 14.3%, being stable for 100 h [4,5,8,16,17]. Despite the high PCE, the CsPbI_3_ QDs solar cells generally are fabricated with a planar architecture, as it turns out to be highly challenging to homogeneously incorporate QDs into the mesoporous structure. At the same time, this approach is difficult to replicate in planar solar cells, since the mesoporous titania is more prone to the infiltration of the chemicals and salts used in this strategy. In planar solar cells architecture, the efficiency exceeded over 14%, with a stability of almost 10 days if the QDs are passivated with a treatment with glycine [18]. Therefore, there is a lack of strategies that limit the use of chemicals and solvents to improve the CsPbI_3_ stability, preserving the efficiency and making it easy to reproduce regardless of the structure used.

In this work, the combined use of solvent and controlled UV treatment is reported. The choice lies in the need of, firstly, removing the ligand in excess and guarantee the QD-to-QD electronic coupling, and secondly, improving the stability of the perovskite to prepare also a material not affected by the further deposition steps. The process combines the desired desorption of the surface ligands and the ion migration, which facilitate the passivation of surface vacancies and improve the stability. Previous reports show that moderate UV light (lamp power 7 W, λ = 365 nm) promotes the oriented attachment of CsPbI_3_ QDs to form large nanoparticles (40 nm) along (110) facets. On the other hand, a higher UV power (lamp power 100 W, λ = 365 nm) induces the degradation of the α-CsPbI_3_ QDs into photoinactive δ-phase and finally into the precursors [19]. The photo-induced attachment has also been proven in CsPbBr_3_ nanoplatelets and nanocubes [20]. Since CsPbBr_3_ is more stable than CsPbI_3_ [21], the latter would require less energy to undergo this process, thus avoiding degradation phenomena. It is worth to note that in systems like CdSe nanoplatelets, light-induced attachment even at 1 W cm^−2^ is not experimented, since its covalent nature present negligible ion mobility [22]. Pointing out that the ion mobility and the presence of vacancies formed after the deposition process and washing treatment are fundamental for the success of the UV treatment. Finally, with this dry approach we obtained more stable materials and solar cells in air, with an increased efficiency up to 7.4%, due, at the same time, to the improved optoelectronic properties and to the interface structural quality, taking advantage from the own photoactivity of the perovskite.

## 2. Materials and Methods

### 2.1. Synthesis of CsPbI_3_ QDs

All the purchased chemicals were used as received. CsPbI_3_ QDs were synthesized according to the hot-injection method described elsewhere [7], by mixing stoichiometrically Cs-oleate and lead iodide (PbI_2_, AB111058, 99.999%, acbr GmbH, Karlsruhe, Germany. Cs-oleate was first prepared by mixing 0.610 g Cs_2_CO_3_ (202126, 99.9%, Sigma-Aldrich, Madrid, Spain), 2.5 mL oleic acid (OA, 364525, 90%, Sigma-Aldrich, Madrid, Spain), and 30 mL of 1-octadecene (ODE, O806, 90%, Sigma-Aldrich, Madrid, Spain) into a 50 mL-three neck flask for 90 min under vacuum. Then, the temperature was increased to 150 °C under N_2_-atmosphere to reach the complete Cs_2_CO_3_ dissolution. For the injection step, Cs-oleate was kept at 120 °C under N_2_-purge. Then, 1 g PbI_2_ and 50 mL ODE were mixed into a 100 mL-three neck flask at 120 °C under vacuum and vigorous stirring for 1 h. A preheated mixture of oleylamine (OLA, HT-OA100, 98%, Sigma-Aldrich, Madrid, Spain) and OA (5 mL each one into a beaker at 130 °C for 30 min) was added under N_2_-purge and temperature was rapidly raised up to reach 170 °C. In this point, preheated Cs-oleate (4 mL) was immediately injected, observing a luminescent red colloidal solution. For the purification step, 60 mL of methyl acetate (MeOAc, 296996, 99.5%, Sigma-Aldrich, Madrid, Spain) with 32 mL of liquor solution are centrifuged at 4700 RPM for 5 min. The precipitated QDs were recovered from supernatant and re-dispersed in 5 mL of hexane (CHROMASOLV, 34859, 99.7%, Honeywell, Barcelona, Spain). A second purification was conducted by adding 5 mL of MeOAc to the QDs dispersion and centrifuged at 4700 RPM for 5 min. The QDs product was obtained and re-dispersed with 10 mL of hexane. The final colloidal solution was stored in the fridge at least for 24 h before use it.

### 2.2. Quantum Dot Solution Preparation

A prepared solution of CsPbI_3_ QDs in hexane was stored at 4 °C into a fridge. After 1 day, the supernatant was taken out and centrifuged at 6500 rpm for 10 min. After drying the new supernatant with nitrogen flow, a proper amount of octane was added to obtain 70 mg/mL.

### 2.3. Solar Cell Fabrication

Metallic zinc powder was used for etching with 6 M HCl. Subsequently, Indium-tin-oxide (ITO) substrates were brushed and then sequentially sonicated in soapy water, milliQ water, acetone, and isopropanol (15 min each solvent). Substrates were dried with an air gun. For the tin oxide layer deposition, the substrates were treated in UV–ozone for 20 min, then 110 µL of 2.6% *v/v* SnO_2_ solution diluted from a 15% tin (IV) oxide colloidal solution (Alfa Aesar, Barcelona, Spain) were spin coated during 40 s at 3000 RPM. Before the annealing for 30 min at 150 °C in a hot plate, one side of the substrates was cleaned with a wet cotton swap around 5 mm to ensure the contact with the gold electrode.

The solar cells were fabricated inside a nitrogen filled glove box with O_2_ and H_2_O level lower than 5 ppm. To deposit the absorber layer, the ITO/SnO_2_ substrates were treated by UV-ozone during 60 min, then 20 µL of 70 mg/mL CsPbI_3_ QDs in octane were spin coated according to the following program: 1 s to reach 1000 RPM, 20 s at 1000 RPM, 1 s to reach 2000 RPM, 85 s at 2000 RPM, and stops in 1 s. Then, 300 µL of ethyl acetate were quickly added when the spin reach 2000 RPM. For the devices treated with UV, every layer after the ethyl acetate washing was put under UV light (ELC 500 UV cure chamber, 9 W UV lamp optical) for 2 and 4 min (this process was also conducted inside the glovebox). This process was repeated to obtain layer by layer the desired thickness of the final film. 2,2′,7,7′-Tetrakis[N,N-di(4-methoxyphenyl)amino]-9,9′-spirobifluorene (Spiro-OMeTAD) was spin coated on top of the perovskite QDs from a fresh Spiro-OMeTAD (Feming) solution according the literature [23]. Finally, 80 nm of gold contacts were thermally evaporated.

### 2.4. Photoelectric Characterization

The photovoltaic devices were characterized using an Abett Solar simulator equipped with an AM 1.5 filter. The light intensity was adjusted to 100 mW cm^−2^ using a calibrated Si photodiode (Hamamatsu S1133). The active area of the cell is 0.121 cm^2^, determined with a metallic external mask. All the J–V measurements were carried out after leaving the devices 1 min under 1 sun illumination in air at a temperature around 25 °C and a relative humidity around 50%, without encapsulation. Each curve was generated using 123 data points from a starting potential of 1.2 V to a final potential of −0.05 V (reverse scan) using a scan rate of 10 mVs^−1^. From the curves were calculated parameters like, the photoconversion efficiency (PCE), the short circuit current density (J_sc_), the maximum current produced for the device when there is no external bias opposing the electrons flow (V = 0), the open circuit voltage (V_oc_), the maximum voltage produced by the cell which can be rationalized as the opposing voltage needed to stop the electrons flow (J_sc_ = 0 mA/cm^2^), and the fill factor (FF), determined as the ratio of the maximum power density produced to the product of V_oc_ and J_sc_ of the solar cell. The incident photons to current efficiency (IPCE) measurements of the encapsulated devices are performed with a QEPVSI-b Oriel measurement system.

### 2.5. Structural Characterization

The morphologies of the samples (ITO/SnO_2_/CsPbI_3_) were carried out with a field emission scanning electron microscope (FEG-SEM) JEOL 3100F) operated at 5 kV. Fourier-transform infrared (FTIR) spectra are collected with a FTIR Equinox 55 (Bruker) with an ATR Pro (Jasco) equipped with a diamond crystal, in standard conditions (in the range 600–4000 cm^−1^). Transmission electron microscopy (TEM) images were taken on W JEOL JEM 1010 transmission electron microscope (Akishima, Tokyo, Japan), using an accelerating voltage of 100 kV, with a resolution of 0.4 nm. The X-ray diffraction (XRD) patterns of the CsPbI_3_ films were recorded using an X-ray diffractometer (D8 Advance, Bruker AXS, Karlsruhe, Germany) (Cu Kα, the wavelength of λ = 1.5406 Å) within the range of 5–80°.

### 2.6. Optical Characterization

The steady state absorption spectra of the perovskite films on glass substrate were achieved by using a UV/Vis absorption spectrophotometer (Varian, Cary 300, Palo Alto, USA), the wavelength range for the measurements was 400–800 nm. The emission measurements (steady state photoluminescence and time resolve photoluminescence) were collected by a Horiba Fluorolog Photoluminescence (PL) and measurements were conducted at an excitation of 532 nm. Since the absorbance at the excitation wavelength was not the same, to make a fair comparison the PL emission was normalized dividing by (1−10^−Abs^), which is usually used to compare quantum yields. All measurements were carried out under ambient conditions.

## 3. Results and Discussion

**Figure 1:**
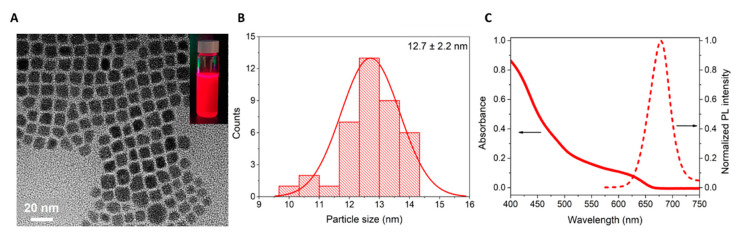
(**A**) TEM image of the CsPbI_3_ quantum dots (QDs) synthetized at 170 °C (inset: CsPbI_3_ QDs solution in octane), (**B**) CsPbI_3_ QDs size distribution histogram, (**C**) absorption and emission spectra of the synthetized CsPbI_3_ QDs in solution.

The CsPbI_3_ QDs used for the preparation of PQDSCs show a nanocube shape, with a size distribution of 12.7 ± 2.2 nm, see Figure 1a,b. The optical properties reveal an emission at 680 nm, see Figure 1c, and a PLQY of 94% and 82% after the first and second purification steps, respectively.

**Figure 2:**
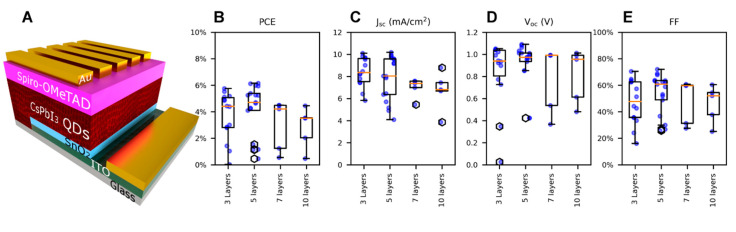
(**A**) Sketch of the perovskite solar cells architecture; photovoltaic statistic (box plot) of the QDs solar cells figures of merit, with different number of layers stacked by spin-coating and ethyl-acetate (EtOAc) washings: (**B**) power conversion efficiency PCE. (**C**) Short circuit current density, J_sc_. (**D**) Open circuit voltage, V_oc_. (**E**) Fill factor, FF.

The deposition of the CsPbI_3_ QDs colloidal solution dispersed in octane, is preliminary optimized to remove the excess of organic ligand coming from the synthesis, by selecting between the methyl acetate and ethyl acetate and by choosing the right volume needed to deposit a second layer without washing the first one (see experimental section) [14]. The CsPbI_3_ film is used as active layer in PQDSCs (ITO/SnO_2_/CsPbI_3_/Spiro-OMeTAD/Au), see Figure 2a, made by depositing layer by layer 3, 5, 7, and 10 layers of QDs. With the aim of depositing one layer on the top of the other, the solvent washing treatment is vital, using EtOAc in our case. In this context, the QDs become insoluble in the solvent in which they are well dispersed (octane) because of the presence of the organic capping ligand. Then, the screening on the thickness is carried out. The photovoltaic parameters of the prepared cells are depicted in Figure 2b–e. The highest photocurrent densities, J_sc_, are achieved with 3 and 5 layers, see Figure 2c. The maximum obtained V_oc_ and fill factor (FF) are almost the same, see Figure 2d,e, respectively. J_sc_ slightly decreases from 3 to 5 layers, see Table 1, while for higher number of layers (7) there is an evident drop in the current, likely due to transport limitations as carriers have to travel longer distances, originated by the residual organic ligand. However, losses at the interfaces or in the bulk, which is associated to the existence of structural defects and inhomogeneity when the thickness is higher, see below, could also contribute to the performance reduction for the thicker layers prepared.

**Figure 3:**
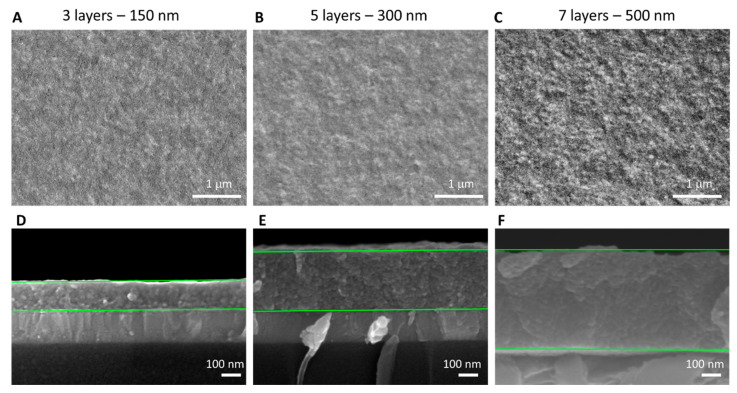
Top view SEM images of the CsPbI_3_ QDs films deposited layer by layer. (**A**) 3 layers, (**B**) 5 layers, (**C**) 7 layers. Cross section SEM images of the staked CsPbI_3_ QDs layers deposited over the SnO_2_ electron transporting layer (ETL). (**D**) 3 layers, (**E**) 5 layers, and (**F**) 7 layers.

The top-view SEM images of the CsPbI_3_ QDs films made of 3, 5, and 7 layers, see Figure 3a–c, respectively, show very similar morphologies, although a minor difference can be noticed for the 7 layers film, Figure 3c, as it seems that relatively small and bright aggregations exist. This fact agrees with the wavy surface observed through the cross-section SEM profile, Figure 3f. In Figure S1, a higher magnification shows the film consisting of closely packed assemblies of discrete QDs, indicating that the QDs film morphology is preserved after each EtOAc washing. From the cross section SEM images, the films thicknesses of 3 and 5 layers were estimated to be 150 nm and 300 nm, respectively, which is in line with the thickness range, between 100–400 nm commonly used in CsPbI_3_ QDs solar cells [4,13]. On the other hand, the 7 layers film has a thickness around 500 nm. All these evidences fully explained the PCE trend by changing the thickness of the active layer. In particular, with 7 layers the surface is undulating and presents small aggregations, see Figure 3c,f, leading to not homogeneous deposition of the Spiro-OMeTAD on the top. The higher light harvesting in a thicker film cannot compensate the deleterious transport and defects, and the J_sc_ obtained is even lower than that of the device with 3 layers, see Figure 2c. Moreover, in line with this observation, the FF also decreases, see Figure 2e.

**Figure 4:**
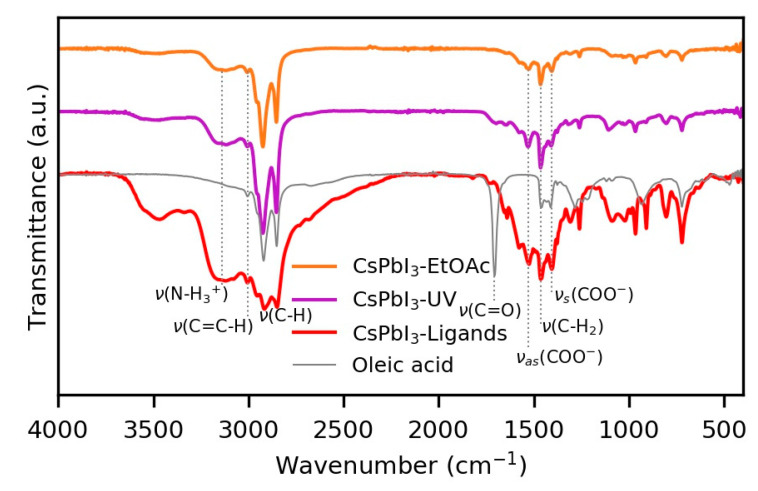
FTIR spectra of neat oleic acid (gray) and spin-coated CsPbI_3_ QDs film (without EtOAc washing of the film): CsPbI_3_ Ligands (red); spin-coated film washed with EtOAc: CsPbI_3_-EtOAc (orange). Spin-coated layer exposed to UV light for 2 min after washing: CsPbI_3_-UV (purple). In all the cases the film constituted of 1 spin-coated layer of QDs.

Attending to the above results, the most suitable condition is obtained for 5-layer devices, even whether the PCE is still low, so a further optimization has done only focusing on the most promising checked configurations, in terms of current densities, 3- and 5-layer devices. In order to increase the carrier transport along the perovskite QDs film, it is necessary the careful control of the ligand density covering the QDs. Oleate and oleylammonium species are removed by washing with various polar solvents. Nevertheless, excessive washing causes Cs^+^ vacancies and strong aggregation or degradation [16,24]. On the other hand, UV/O_3_ has proven to remove ligands from CsPbBr_3_ QDs and simultaneously increase the PL emission, by a photoactivation mechanism [25]. The QDs surface is reduced of ligands, and subsequently photo-oxidized and smoothened upon exposure to UV in air, with positive consequences, for instance, on the light emitting diodes performances [25]. Previous studies reported that no significant photo-activation is observed in a dry inert N_2_ atmosphere. This fact points that photo-activation is triggered mainly due to photo-oxidation [26]. Considering that the black phase of the CsPbI_3_ is not stable in air, we explored the use of the UV light under nitrogen atmosphere to cure the CsPbI_3_ QDs after the organic ligand partial removal, as a tool to improve the solar cell efficiencies, avoiding the detrimental effect of the oxygen or of the wet chemistry. A comparative analysis between the CsPbI_3_ films after different treatments to partially remove the organic ligand is carried out. The common washing with EtOAc and the UV treatment are used with the intention of dissolving and partial removing selectively the organic capping ligands. Figure 4 shows the FTIR spectrum of liquid neat oleic acid, with its characteristic carboxylic acid stretching mode, ν (C = O) at 1708 cm^−1^ [27]. This peak is not observed in the films, see Figure 4, indicating that this ligand is present as oleate species on the QDs. This is confirmed by the signals of the COO^−^ group of the oleate [28], the symmetric and asymmetric stretching modes at 1409 and 1530 cm^−1^, respectively. Oleylammonium can be spotted by a broad resonance centered at 3138 cm^−1^ corresponding to vibrations of ammonium groups, ν (N−H_3_^+^) [14]. The peaks at ν (C−H) = 2780−3000 cm^−1^, ν (C = C−H) = 3005 cm^−1^, and ν (C−H_2_) = 1466 cm^−1^ are also related to the long alkyl chain of the oleic species or to the acetic acid formed in situ.

The signals intensity of the film washed with EtOAc are decreased, compared with the signals of the sample without washing (CsPbI_3_-Ligands), indicating the partial removal of organic ligands [24]. In the case of the sample subjected to UV treatment, there is no proof of substantial ligand removal induced by the UV light, as there is no significant difference in the signals intensity, thus the ligand surface density of the QDs is roughly the same. Note that with the FTIR analysis we confirm that the organic species present in solution interact with the QDs also after the UV treatment and that if the UV could be an efficient way to remove organic ligand in air under nitrogen atmosphere the FTIR shows no signs of further removal. However, the efficiency of the devices made with the UV treatment has signs to be different, which as explained below will be due to an alternative mechanism, which in this study is not ascribed to the amount of ligand coordinated with the QDs.

**Figure 5:**
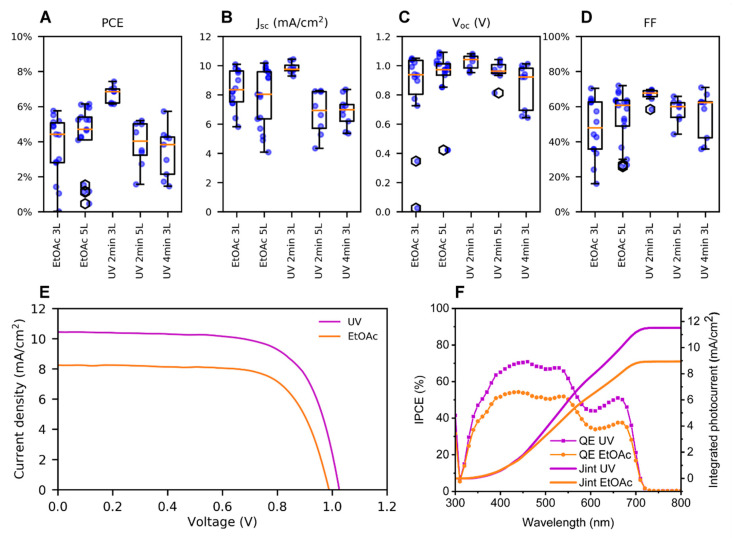
Photovoltaic parameters of the CsPbI_3_ perovskite QDs solar cells with different number of layers and UV treatments. EtOAc is the sample in which only the EtOAc washing is used. UV means that each layer was treated with the UV under nitrogen after every washing. Box plots for (**A**) power conversion efficiency, PCE. (**B**) Short circuit current density, J_sc_. (**C**) Open circuit voltage, V_oc_. (**D**) Fill factor, FF. (**E**) J–V curves of the best solar cells employing 3 layers of CsPbI_3_ QDs stacked by washing (EtOAc) and washing + UV treatment. (**F**) IPCE curves of encapsulated solar cells with 3 layers of CsPbI_3_ QDs as light absorber with and without UV treatment.

Figure 5 shows the statistic of the figures of merit and refers to the devices made with 3 or 5 QDs layers. In the reference devices, every spin-coated layer is washed with EtOAc to partially remove the capping ligands from the QDs surface. When the semi-devices undergoing UV treatment are put under UV radiation for 2 min after every EtOAc washing, it is found that the J_sc_, the V_oc_, the FF, and especially the reproducibility of the devices significantly improves, see Figure 5 and Table 2. A UV exposure less than 2 min leads to the partial detachment of the layer after EtOAc washing, Figure S2.

**Figure 6:**
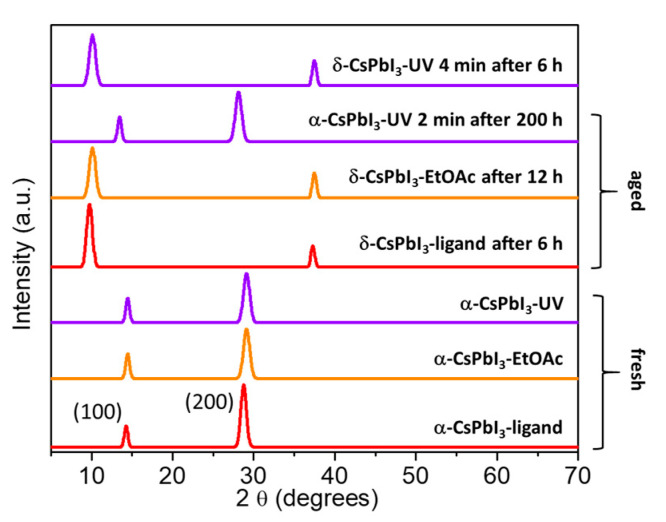
XRD patterns of the CsPbI_3_-ligand (1 layer), CsPbI_3_-EtOAc (3 layers), and UV treated (3 layers) fresh samples and after 7 days in air; different UV exposure times (2 min and 4 min) are reported.

It is also worth to note that after air exposure during J/V characterization, the solar cells without UV treatment quickly turned yellow (Figure S2b). The yellow color is due to phase transformation from black α-CsPbI_3_ to yellow δ-CsPbI_3_, as we have analyzed by X-ray diffraction (XRD), see Figure 6. By contrast, UV treated samples are stable at least during 7 days at 25% of relative humidity, see Figure 6. X-ray diffraction patterns of the CsPbI_3_ films confirm that fresh samples always present black α-CsPbI_3_ independently of the treatment. After 6 h, these films are not more stable and the peak corresponding to the yellow CsPbI_3_ δ-phase at 9.8° appears. However, the films treated with 2 min UV are stable at least for 7 days, as it has been previously commented. Interestingly, a too long UV treatment (4 min) cannot avoid the phase transformation into the δ-phase after 6 h [29].

On the other hand, by comparing 5-layer devices with and without UV treatment (total exposure time of 10 min), a decrease in the J_sc_ is observed. In line with this, the 3-layer devices exposed for 12 min to the UV (4 min for each layer) also result in a decrease in the J_sc_. The V_oc_ and FF increase even after this long exposure for both cases, but the negative effect in the current predominates and the efficiency decreases (Figure 5c,d and Table 2). Lead halide perovskites in fact suffer an aggressive degradation after prolonged UV exposure [20,30,31]. This fact indicates that the total UV exposure times longer than 10 min generate photodegradation in the perovskite, as demonstrated with the XRD measurements, Figure 6. However, reduced time UV treatments can significantly contribute to the device performance and stability. In brief, the best PCE obtained, 7.4% for the solar cells based on 3 layers treated with the UV, originated from the higher J_sc_ and V_oc_ than the samples without UV, and from the higher FF than the sample with more layers, see Table 2. The incident photon-to-electron conversion efficiency (IPCE) characterization, see Figure 5f, confirms the presence of black phase of CsPbI_3_ in working encapsulated PQDSCs with a good agreement between the photocurrents calculated by this method and the measured by J–V curves, see Table S1, where the higher current observed in the IPCE could be due to the encapsulation of both the devices. The encapsulation was done to untangle the increased J_sc_ from the degradation observed in the non-treated devices, as it will be discussed later. The IPCE characterization relates also to many factors such as the band gap of the photoactive material [32,33,34], the light that goes through the transparent electrodes and the effectiveness of charge collection at certain wavelength [32,35]. The shape of the IPCE spectra is consistent with previous work, reporting direct planar solar cells architecture, where the onset at short wavelengths is related to light absorbance from the ITO glass and SnO_2_ [18] and the one at the long wavelengths is at 680 nm, below the onset around 700 nm of solar cells employing bulk CsPbI_3_. This demonstrates the preservation of the quantum confinement for both non-treated and UV treated devices [4,15,36,37].

**Figure 7:**
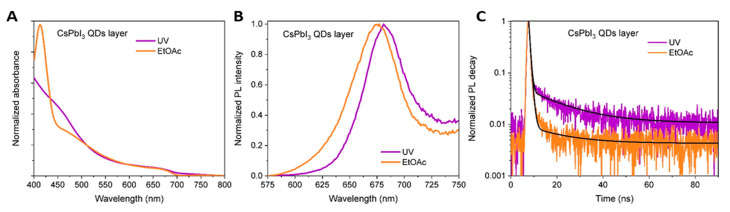
Optical characterization of CsPbI_3_ QDs 3 stacked layers deposited on glass with and without UV treatment. (**A**) Normalized absorption spectra, (**B**) steady state PL emission spectra, excitation wavelength 530 nm, and (**C**) time resolved PL decay fitted whit a biexponential decay function.

The optical properties are analyzed in Figure 7. The absorption spectra, see Figure 7a, of the untreated film confirms, in accordance with the XRD (Figure 6), the fast degradation of the sample without UV treatment, as the peak around 416 nm evidences the presence of CsPbI_3_ yellow phase (δ-CsPbI_3_) [38]. Moreover, there is no change in the absorbance intensity at the onset, so the UV does not affect the layer thickness. The photoluminescence (PL) spectra show a blue shift from 680 to 675 and an increase in the full width half maximum (FWHM) from 51 nm to 57 nm (Figure 7 b) in line with the underway degradation of the CsPbI_3_-EtOAc. In addition, it has been found that after the UV treatment, QDs have longer PL time decay with electron lifetime, see Figure 7c, going from 1.9 ns to 11.8 ns, Table S2, representative of the surface emission trap concentration reduction [39]. This behavior can be attributed to either long carrier diffusion length in bigger CsPbI_3_ domains, as in the case of CsPbBr_3_ [20], or to the passivation of the defects and a lower non-radiative recombination processes. The enlargement of the QDs size is not corroborated, as the PL emission of the CsPbI_3_-UV is at the same wavelength of the original CsPbI_3_, see Figure 1. Thus, the only mechanism plausible to explain the longer time decay remains the decrease of the non-radiative recombination. Accordingly, despite the same thickness of the layers with and without UV treatment, the J_sc_ of the devices based on the material treated with UV is higher. The slow and constant degradation of the samples without UV also can contribute to a lower current value, but the preservation of the bandgap observed in the IPCE for encapsulated device clearly demonstrated that the degradation process is not the main cause of the lower J_sc_. Instead, the reduction of the non-radiative recombination is correlated with the improved solar cells.

**Scheme 1:**
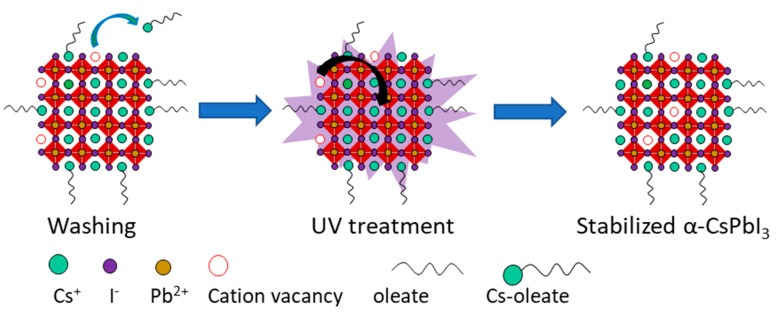
Sketch of the photo induced phase stabilization of CsPbI_3_ QDs: At first, surface cation vacancies are created during de EtOAc washing by the removal of Cs-oleate. Surface cation vacancies are detrimental for the phase stability. The UV exposure enhances ionic mobility and the cation vacancies diffuse to the QD bulk stabilizing the cubic phase.

Finally, the most notable difference of the short duration UV treatment is the improved ambient stability of the QDs films. We propose a mechanism to understand this behavior, summarized in Scheme 1. It is well known that the surface vacancies would generate substantial dangling bonds absorbing moisture that accelerate the phase transition [36]. Since the surface vacancies in the QDs with and without UV treatment are not determined by difference in the chemical surrounding, as we have demonstrated by FTIR and since inside the glovebox photo-oxidation unlikely occurs, the rise of the observed improved stability is explained through another mechanism. It has been demonstrated that the α- and δ-phases internal energy difference decreases upon creation of cation vacancies defects [40]. They have a higher formation energies in δ-phase [40] while in the α-phase, the organic ligands coordinate the cations and create Cs-oleate and Pb-oleate pairs, by extracting metal cations from the surface and leave cation vacancies, according to the nanosizing effect [16,40]. These vacancies diffuse into the QDs bulk due to the short migration path that requires the passage of metal cations from the center of the QDs to the surface, passivating superficial vacancies and reducing the surface recombination centers, see Scheme 1 [40]. Following the postulated above, a possible explanation for the increased stability is as follows. Cs^+^ vacancies are created by EtOAc washing that carries some of the bound ligands (Cs-oleate) off the surface. Vacancies created are, in theory, detrimental to the stability of QDs, as is the case of the UV-free films. In the case of UV treatment, the increased ion mobility in perovskite materials [22,41,42,43] facilitates the migration of cations from the interior of the QDs to the surface (Scheme 1), in line with the mechanism postulated by Y. Kye et al. for which the presence of cation vacancies in the QD bulk stabilizes α-CsPbI_3_ [40].

## 4. Conclusions

In conclusion, a time limited UV treatment is successfully used to improve the stability of the CsPbI_3_ QDs. The untreated material shows signs of degradation both during the spin-coating of the layer on top and always in air and in turn the untreated device degrades after exposure to air, as demonstrated by the presence of photoinactive δ-CsPbI_3_. The improved current and PCE for the device with the short UV treatment is related with a more air stable material, facile and robust interface optimization, and decreased non-radiative recombination. Finally, a material stable for 200 h and devices with a higher reproducible efficiency of the 7.4% have been obtained, demonstrating that the control of cation vacancies without using the wet chemistry is a promising route for the improvement of the perovskite QDs solar cells.

## Figures and Tables

**Table 1:** Mean and standard deviation (SD) for the photovoltaic figures of merit of devices with different number of QDs layers.

Number of Layers	PCE (%) (Mean ± SD)	J_sc_ (mA/cm^2^) (Mean ± SD)	V_oc_ (V) (Mean ± SD)	FF (%) (Mean ± SD)
3	3.7 ± 1.8	8.4 ± 1.3	0.840 ± 0.290	47.4 ± 16.2
5	4.2 ± 1.9	7.9 ± 2.0	0.921 ± 0.192	53.9 ± 14.7
7	3.0 ± 1.7	7.0 ± 0.8	0.776 ± 0.270	47.9 ± 15.1
10	2.8 ± 1.4	6.7 ± 1.6	0.810 ± 0.220	46.0 ± 12.8

**Table 2:** Mean and standard deviation (SD) for the photovoltaic figures of merit of devices with different number of QDs layers.

Variation	PCE (%) (Mean ± SD)	Jsc (mA/cm^2^) (Mean ± SD)	Voc (V) (Mean ± SD)	FF (%) (Mean ± SD)	Best PCE (%)
EtOAc 3 L	3.7 ± 1.8	8.4 ± 1.3	0.840 ± 0.290	47.4 ± 16.2	5.8
EtOAc 5 L	4.2 ± 1.9	7.9 ± 2.0	0.921 ± 0.192	53.9 ± 14.7	6.2
UV 2 min 3 L	6.7 ± 0.4	9.8 ± 0.4	1.025 ± 0.047	66.6 ± 3.5	7.4
UV 2 min 5 L	3.9 ± 1.2	6.8 ± 1.4	0.961 ± 0.066	57.7 ± 6.5	5.2
UV 4 min 3 L	3.4 ± 1.3	6.9 ± 1.0	0.864 ± 0.145	55.4 ± 12.7	5.7

## Supplementary Materials

The following are available online at https://www.mdpi.com/2079-4991/10/8/1586/s1, Figure S1: Top view SEM image of the CsPbI_3_ QDs 3 layer film with higher magnification, Figure S2: (a) Photograph of accidental detachment of photoactive layer after EtOAc washing in a device with 1 min UV treatment. (b) Pictures of the solar cells after air exposure, left EtOAc and right UV treated device, Table S1: Photovoltaic parameters extracted from J–Vs scans, best device of each variation, Table S2: Time resolved PL decay fitting parameters with a biexponential function.
