# Supplementary material for: Photo-Induced Black Phase Stabilization of CsPbI3 QDs Films
Source: Nanomaterials (Basel). 2020 Aug 12;10(8):1586. doi: 10.3390/nano10081586 (PMC7466586; doi:10.3390/nano10081586)
Supplement: Supplementary file 1 [file nanomaterials-10-01586-s001.pdf]

nanomaterials

Article

Photo-Induced Black Phase Stabilization of $CsPbI_3$ QDs Films

Eider A. Erazo [1,2], H.E. Sánchez-Godoy [1,3], Andrés F. Gualdrón-Reyes [1], Sofia Masi [1,*] and Iván Mora-Seró [1,*]

1 Institute of Advanced Materials (INAM), Universitat Jaume I (UJI), Avenida de Vicent Sos Baynat, s/n, 12071 Castellón de la Plana, Spain; ea.erazo@uniandes.edu.co (E.A.E.); Humberto.Sanchez@academico.udg.mx (H.E.S.-G.); gualdron@uji.es (A.F.G.-R.)
2 Departamento de Química, Universidad de los Andes, Bogotá D.C. 111711, Colombia
3 Universidad de Guadalajara, Centro Universitario de los Lagos, Lagos de Moreno, Jalisco, C.P. 47460, Mexico
* Correspondence: masi@uji.es (S.M.); sero@uji.es (I.M.-S.)

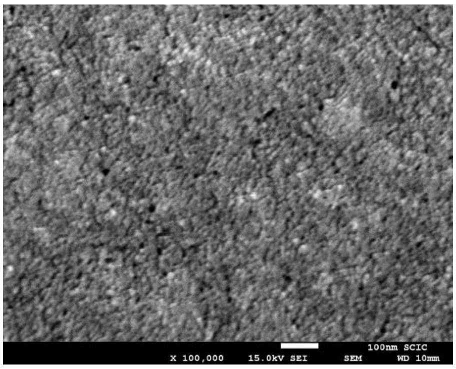

Figure S1. Top view SEM image of the $CsPbI_3$ QDs 3 layer film with higher magnification.

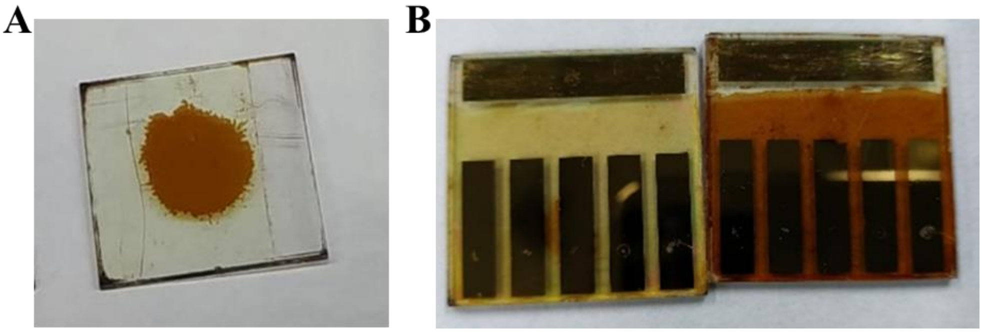

Figure S2. (**a**) Picture of accidental detachment of photoactive layer after EtOAc washing in a device with 1 min UV treatment. (**b**) Pictures of the solar cells after air exposure, left EtOAc and right UV treated device.

Table S1. Photovoltaic parameters extracted from J–Vs scans, best device of each variation.

Variation	Jsc (mA/cm²)	V_{oc} (V)	FF (%)	PCE (%)
EtOAc 3 layers	8.2	0.992	70.5	5.8
EtOAc 5 layers	9.6	1.011	63.6	6.2
UV 2 min 3 layers	10.4	1.031	69.0	7.4
UV 2 min 5 layers	8.3	1.041	60.2	5.2
UV 4 min 3 layers	8.2	0.982	70.9	5.7

Table S2. Time resolved PL decay fitting parameters with a biexponential function.

Sample	τ_1 (ns)	B_1 (%)	τ_2 (ns)	B_2 (%)	τ_{ave} (ns)
$CsPbI_3$-EtOAc	15.5	8.4	0.65	91.6	1.9
$CsPbI_3$-UV	25.0	46	0.57	54	11.8

© 2020 by the authors. Licensee MDPI, Basel, Switzerland. This article is an open access article distributed under the terms and conditions of the Creative Commons Attribution (CC BY) license (http://creativecommons.org/licenses/by/4.0/).
